# Successful Management of Meralgia Paresthetica by Hydro Dissection in a Middle-Aged Male: A Case Report

**DOI:** 10.7759/cureus.25945

**Published:** 2022-06-14

**Authors:** Jeshnu Tople, Vidur Mago, Vivek Chakole, Yatharth Bharadwaj

**Affiliations:** 1 Anaesthesiology, Jawaharlal Nehru Medical College, Wardha, IND; 2 Department of Anesthesia, Jawaharlal Nehru Medical College, Datta Meghe Institute of Medical Sciences, Wardha, IND

**Keywords:** usg guided procedures, paresthesia, local anaesthesia, mononeuropthy, nerve hydrodissection, meralgia paresthetica

## Abstract

Meralgia paresthetica (MP) comes under a wide array of syndromes known as Mononeuropathies, wherein the subject presents symptoms of tingling burning pain as well as numbness in the thigh’s lateral aspect which occurs as a result of the lateral femoral cutaneous nerve being compressed. The condition is commonly seen in association with diabetes mellitus and obesity. The nerve’s anatomy which involves tunnelling through the inguinal ligament predisposes it to get entrapped. However, the pathophysiology of this condition additionally involves inflammation other than the usual entrapment neuropathy. Its characteristic clinical presentation usually clinches the diagnosis and the prognosis is generally favourable with the use of multiple modalities of treatment but not limited to peripheral nerve blocks, nerve decompression, neurectomy as well as pulsed radiofrequency neuromodulation. We present a case of MP which was treated promptly with hydro dissection in a rural tertiary care hospital in central India.

## Introduction

Mononeuropathies are not uncommon conditions in middle age groups from 45 to 65 years old with an incidence of 0.9-1% [1]. Among these meralgia paresthetica (MP) has an average incidence of 4.3 in 10,000 patients years [2] with higher predilection seen in adult females. The condition is commonly seen in association with diabetes mellitus and obesity [3]. It is primarily characterized by a tingling sensation, numbness and burning pain seen over the antero-lateral aspect of the ipsilateral thigh. It is caused by entrapment and compression of the lateral cutaneous nerve which is also called the lateral femoral nerve which arises from the dorsal root of L2-L3 from the spinal cord and reaches up to the thigh.

## Case presentation

A 46-year-old male presented with a complaint of tingling and burning pain over the antero-lateral aspect of the right thigh for six years. The patient had no history of hypertension/diabetes mellitus/tuberculosis/thyroid/asthma. There was no history of any substance abuse such as alcoholism, cigarette smoking, or tobacco chewing. Vitals were within normal range, Systemic examination was normal apart from pain over the thigh. The condition affected his day-to-day lifestyle as he is a driver by profession. The patient consulted numerous general physicians, various specialists and underwent multiple modalities such as physiotherapy, ayurvedic treatment, etc. which were ineffective. The patient then came to our pain clinic where after excluding other differentials such as spinal nerve radiculopathy at L1-L3, chronic appendicitis, malignancy, or metastasis at the iliac crest on an ultrasound examination it was found that the lateral cutaneous nerve of the thigh on the left side was thickened as compared to the contra-lateral side along with paresthesia, for which diagnosis of MP was made. Due to the neuronal origin of the pain, the patient was started on tab gabapentin 300 mg TDS (thrice a day) and nortriptyline 10 mg HS but this treatment was ineffective. The patient was then counselled, and he consented to nerve hydro dissection with local anaesthetic (LA) and steroid injection.

**Figure 1:**
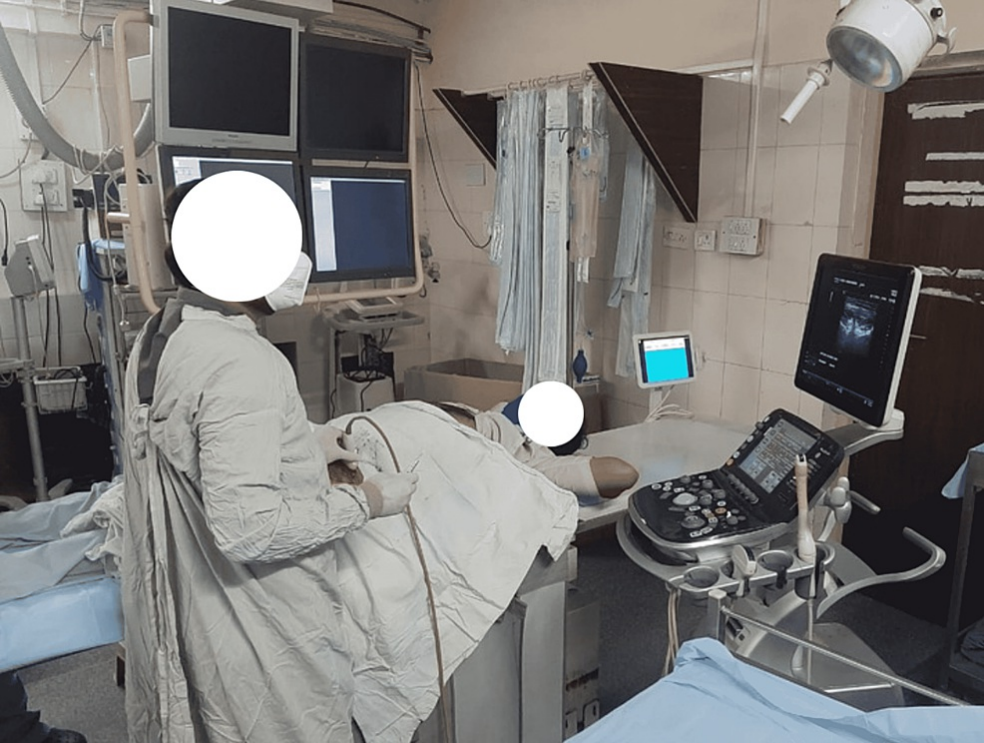
USG guided Nerve Hydro-Dissection.

**Figure 2:**
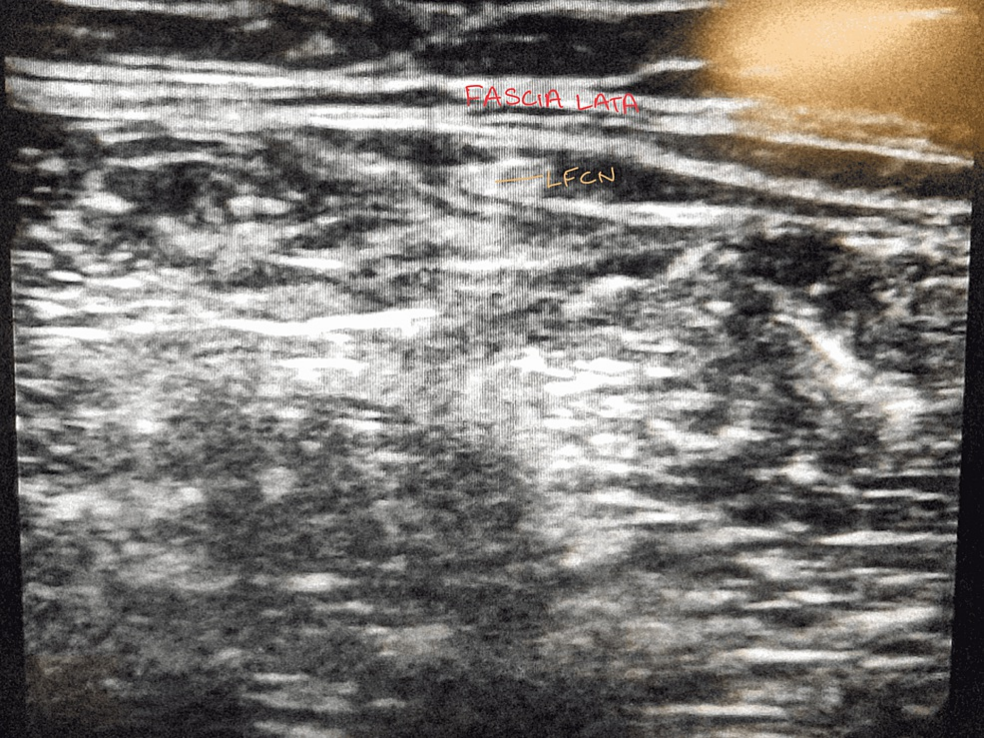
Ultrasonography showing the procedure of hydro dissection of lateral cutaneous nerve of thigh

Preoperative history and examination were done thoroughly. The procedure was explained in detail along with the risks involved and written informed consent was taken. Preoperatively lidocaine sensitivity test was done. The patient was brought to the sterile procedure room, and monitors were attached to record heart rate, blood pressure, and oxygen saturation, and vitals were noted. The intravenous (IV) cannula was secured in case of any reaction to the local anaesthetic drug, normal saline was started. Under all aseptic conditions, as depicted in Figure 1, hydro dissection of the lateral cutaneous nerve (LFCN) was done under ultrasound guidance after identifying the nerve using 20 ml of 1% lignocaine with particulate methylprednisolone 20 mg (Figure 2).

The patient had immediate pain relief with mild numbness which lasted for 1.5 hours. The numeric rating scale (NRS) score on the pain detection tool was 5. The patient was reviewed on basis of a numeric rating scale with 1 being mild and 10 being severe pain, on day 15, the NRS score was 1/10, on day 30 it was 1/10, at 3 months it was 1/10, and at 6 months it was 2/10. Hence providing efficient and prolonged pain relief. 

## Discussion

Medications such as tricyclic antidepressants (amitryptyline) and anticonvulsants (gabapentin), among others are commonly used drugs to relieve pain in chronic neuropathic conditions, but these have significant side effects such as sedation. Pulse radio frequency ablation and surgical resection are just two of the therapy options for MP. Conservative methods such as anti-neuropathic medicines such as gabapentin and even narcotics (tramadol) were attempted on our patient, but they only provided brief relief. The patient was given the choice of nerve hydro dissection [4] based on previous studies also patient did not want to undergo any kind of surgical treatment. In nerve hydro dissection the nerve is identified using landmark technique or with USG guidance and an LA agent is injected around the nerve to separate it from its surrounding structure hence reliving from nerve entrapment and also an injection of steroid helps in reducing inflammation. With the help of nerve hydro dissection in our patient, there was a remarkable reduction in pain and overall improvement in health. After nerve hydro dissection, the patient had more than 50% pain alleviation and an improvement in physical health qualify of life. After the treatment, the patient was able to resume their daily routine. Patient’s reliance on previous medications was also reduced and tapered off.

There is no risk of motor harm from the use of medications such as LA and methyl prednisone because LFCN is solely a sensory nerve. Furthermore, using ultrasound, the nerve may be easily located, and the spread of medication can be plainly detected [5] ensuring correct needle placement, and reducing the risk of complications. Structures such as the femoral nerve, femoral artery and bowel can be avoided as well as spills into the distance. Despite the fact that the patient experienced numbness and hypoesthesia, he did not experience much discomfort.

When conservative therapies fail to relive intractable MP, nerve dissection appears to be a viable choice. This is a procedure that is both effective and gives long-term pain relief. Though this procedure has certain limitations such as expertise is required for use of USG and also involves the risk of injection to neighbouring structures but with proper technical know-how it can be a great alternative to surgical intervention.

## Conclusions

This case report demonstrates that ultrasound-guided LFCN nerve hydro-dissection with a local anaesthetic agent and steroid is a safe and effective procedure for patients with intractable MP, USG-guided nerve hydro dissection can provide effective and long-term pain relief and reduces the requirement for oral medication, which have severe systemic side effects such as nephropathy, dependence, and gastritis. We advocate this technique, but it requires more robust evidence and meta-analysis.
